# Meetings of Societies

**Published:** 1935

**Authors:** 


### Meetings of Societies

### Bristol Medico-Chirurgical Society

SIXTY-THIRD SESSION, 1935-36.

THE Annual Meeting of the Society was held at the University on Wednesday, 9th October, 1935, at 8.30 p.m.; seventy-one members were present. Professor A. Rendle Short resigned the Chair to the new President, Mr. A. E. Iles, O.B.E., F.R.C.S. A vote of thanks to the retiring President was proposed by Mr. Chitty, seconded by Mr. Wood, and carried with acclamation.

Mr. Iles then read his Presidential Address, entitled "**Photography and Medicine,**" which will be published in the next issue. Mr. E. Watson-Williams proposed and Dr. A. L. Taylor seconded a vote of thanks to the President for his address.

The annual reports of the Honorary Secretary, of the Honorary Treasurer and of the Editor of the Society's *Journal* were read and adopted. The Honorary Treasurer suggested that the financial condition of the Society and the number of members would justify a reduction of the annual subscription of members to one guinea.

The Society then elected the officers for the ensuing year, Dr. Richard Clarke being adopted as President-Elect. Mr. Iles kindly consented to serve again as Honorary Treasurer. Mr. R. V. Cooke was elected Honorary Secretary. A very cordial vote of thanks was accorded to Mr. H. L. Shepherd on his retirement from the Honorary Secretaryship, the duties of which post he had so ably discharged during the past five years.

---

The second meeting of the session was held at the University on Wednesday, 13th November, the President in the Chair; forty-eight members were present. Dr. Mayes showed skiagrams and Dr. Fraser pathological specimens.

Dr. George Parker was elected an Honorary Member of the Society, Dr. Nixon voicing the thanks of the Society to

### BRISTOL MEDICO-CHIRURGICAL SOCIETY.

### INCOME AND EXPENDITURE ACCOUNT for the year ended 30th September, 1935.

### INCOME.

| | £ | s. | d. | £ | s. | d. |
|---|---|---|---|---|---|---|
| Members' Subscriptions .. | 306 | 10 | 6 | | | |
| Do., outstanding for 1934–1935 .. .. .. .. .. | 31 | 10 | 0 | | | |
| | | | | 338 | 0 | 6 |
| Interest on Bank Deposits .. .. | | | | 2 | 9 | 9 |
| Interest on £500 4 per cent. Consolidated Stock, less tax .. .. | | | | 15 | 10 | 0 |
| | | | | £356 | 0 | 3 |

### EXPENDITURE.

| | £ | s. | d. | £ | s. | d. | £ | s. | d. |
|---|---|---|---|---|---|---|---|---|---|
| Grant to *The Bristol Medico-Chirurgical Journal* .. .. .. | | | | | | | 150 | 0 | 0 |
| Librarian—Honorarium .. .. .. | | | | | | | 15 | 0 | 0 |
| Miscellaneous Expenses— | | | | | | | | | |
| Goodway–Refreshments | | | | 16 | 16 | 0 | | | |
| Postages and Sundries | | | | 9 | 4 | 0 | | | |
| Printing and Stationery | | | | 9 | 9 | 0 | | | |
| Audit Fee .. .. .. .. | | | | 2 | 2 | 0 | | | |
| Lantern and Cinematograph at Meetings .. | | | | 4 | 4 | 0 | | | |
| University Marshal .. | | | | 1 | 0 | 0 | | | |
| Lewis's Medical and Scientific Library— | | | | | | | | | |
| Subscription | 5 | 10 | 0 | | | | | | |
| Postages .. | 0 | 15 | 7 | | | | | | |
| | | | | 6 | 5 | 7 | | | |
| | | | | | | | 49 | 0 | 7 |
| | | | | | | | 214 | 0 | 7 |
| Balance, being excess of Income over Expenditure for the year, carried to Balance Sheet .. .. | | | | | | | 141 | 19 | 8 |
| | | | | | | | £356 | 0 | 3 |

### BALANCE SHEET as at 30th September, 1935.

### LIABILITIES AND CAPITAL.

| | £ | s. | d. | £ | s. | d. |
|---|---|---|---|---|---|---|
| Sundry Creditors .. .. | | | | 6 | 12 | 0 |
| Capital as at 30th Sept., 1934 .. .. .. .. .. | 747 | 16 | 10 | | | |
| *Add* Surplus for the year on Income and Expenditure Account .. .. | 141 | 19 | 8 | | | |
| | | | | 889 | 16 | 6 |
| | | | | £896 | 8 | 6 |

### ASSETS.

| | £ | s. | d. | £ | s. | d. |
|---|---|---|---|---|---|---|
| Cash at Bank— | | | | | | |
| Current Account .. .. | 258 | 17 | 1 | | | |
| Deposit Account .. .. | 168 | 12 | 5 | | | |
| | | | | 427 | 9 | 6 |
| £500 4 per cent. Consolidated Stock at cost .. .. .. .. .. .. | | | | 437 | 9 | 0 |
| Sundry Debtors—Members' Subscriptions for 1934-1935 outstanding .. | | | | 31 | 10 | 0 |
| | | | | £896 | 8 | 6 |

Audited and found correct,

H. E. KEELER,

*Chartered Accountant,*

*Auditor.*

16–18 Clare Street, Bristol 1.

*7th October,* 1935.

Dr. Parker for his long and valuable services. The proposal to reduce the annual subscription to one guinea was carried. It was resolved to send a subscription of ten guineas to the Royal Medical Benevolent Fund.

Dr. G. de C. Rudolf then read his paper on "**Results of the Treatment of Mental Conditions,**" published on page 219 of this issue. In the ensuing discussion the President, Mr. Walters, Mr. Kemm, Dr. Cookson, Dr. Sutton and Dr. Orr-Ewing took part, and Dr. Rudolf replied.

---

The third meeting of the session was held at the University on Wednesday, 11th December, the President in the Chair; sixty-four members were present. Dr. Bergin showed skiagrams, and Dr. A. L. Taylor pathological specimens. Mr. E. Watson-Williams demonstrated an interesting case of cerebral abscess occurring in a child.*

The alteration in the rules was confirmed, by which the annual subscription is reduced to one guinea from October, 1936.

Professor T. B. Davie then read a paper on "**Intra-cranial Tumours.**" The following took part in the ensuing discussion: Dr. Charles, Dr. Bush, Dr. Rudolf and Mr. Wright.

---

### Radium Therapists Visiting Club.

THE Bi-Annual Meeting of the Radium Therapists Visiting Club was held in Bristol on 26th and 27th October, 1935. The proceedings commenced with an informal lunch at the Elmdale Hotel. This was followed by scientific meetings at the Bristol Royal Infirmary, where papers were read by Dr. A. T. Todd on "**Selenide Treatment of Cancer,**" by Mr. E. Watson-Williams on "**Malignant Disease of the Nasal Accessory Sinuses,**" and by Professor Rendle Short on "**Radium Treatment of Carcinoma of the Bladder and Prostate.**" Cases were shown and an interesting discussion followed each paper. The Radium Officer, by the courtesy of the various members of the Honorary Staff, showed some cases of tuberculous skin lesion treated with radium. The Committee of the Bristol Royal Infirmary entertained the Club and the various speakers to tea.

Sunday morning was devoted to the business meeting and a discussion on the "**Relative Advantages of Gamma Rays of Radium and Deep X-rays.**"

* See *British Medical Journal*, 1935, ii., Dec. 14th, p. 1,152.

### Extract from Editor's Report.

REVIEWS OF BOOKS.

FOR some years past there has been difficulty in obtaining the services of reviewers for the books sent to the *Journal.* All books received by the *Journal* are placed on the shelves of the Medical Library, and in this way the authors obtain a wider publicity and members of the Society have the advantage of seeing a large number of books as soon as they are published. It is important, therefore, that books shall be promptly and adequately reviewed. The Editorial Committee would be very glad to hear from any members of the Society that they are willing to undertake the reviews of books of any branch of the Medical Sciences in which they may be interested.

